# Incidence, Management, and Outcomes of Chylothorax after Lung Transplantation: A Single-center Experience

**DOI:** 10.7759/cureus.5190

**Published:** 2019-07-22

**Authors:** Samuel Jacob, Axel Meneses, Kevin Landolfo, Magdy El-Sayed Ahmed, Ian A Makey, Si M Pham, Mathew Thomas

**Affiliations:** 1 Cardiothoracic Surgery, Mayo Clinic, Jacksonville, USA; 2 Cardiothoacic Surgery, Mayo Clinic, Jacksonville, USA; 3 Cardiothoraic Surgery, Mayo Clinic, Jacksonville, USA

**Keywords:** lung transplantation, chylothorax, complications, management

## Abstract

Background

The objective of this study was to determine the incidence and outcomes of chylothorax after lung transplantation.

Methods

We conducted a retrospective review of our institutional lung transplant registry of 504 adult transplantations done from 2001 to 2015 and identified seven patients (1.38%) with chylothorax. Electronic health records were then analyzed to determine demographics, indications for surgery, management, and outcomes. Survival curves were plotted using the Kaplan-Meier method.

Results

Chylothorax presented in the first week in four (62.5%) patients, and approximately one month later in the remaining three. Nonsurgical management was initially attempted in all patients and succeeded in three (42.9%). Elective surgical ligation of the thoracic duct (LTD) was successful in two (66.7%) out of three patients in whom it was performed. One patient required emergent reoperation for clamshell thoracotomy dehiscence from severe chylothorax. Thoracic duct embolization was attempted but unsuccessful in two patients. Subsequently, one of these patients received a peritoneal-venous shunt and the other underwent LTD. Chylothorax permanently resolved in six patients (85.7%). There were no mortalities directly related to chylothorax. The median time to resolution was 11 days (range: 7-60). The mean survival in months for chylothorax patients was 29.2 (SE 3.1) and 78.2 (SE 2.9) for the remaining patients (*p* = 0.37). The median survival was not reached for the chylothorax group and was 71.8 months (95% CI: 58.0-83.9) for the rest.

Conclusion

Chylothorax is rare after lung transplantation but can lead to major comorbidities and prolonged hospital stay. In our experience, nonsurgical management was successful in up to 40% of patients. LTD should be considered in those who fail conservative management.

## Introduction

A high-output chylothorax is a therapeutic challenge as well as a life-threating complication secondary to persistent drainage of lymph fluids, resulting in immune suppression, malnutrition and electrolytes abnormalities. Although 49% to 54% of the chylothorax is caused by surgery or an invasive procedure, the reported overall incidence of postoperative chylothorax remains very low [1-2]. In an analysis of over 11,000 patients who had general thoracic procedures done at the Mayo Clinic in Minnesota, only 0.42% developed chylothorax, while another study from the University of Alabama reported an incidence of 1.4% after lung resection and mediastinal lymph node dissection [3-4]. The exact incidence of chylothorax after lung transplantation has not been studied in large populations but has been reported in the range of <1% to 11% in smaller cohorts that included some heart-lung transplant recipients [5-8]. Our study was designed to determine the incidence, management, and outcomes of post-operative chylothorax in lung transplant recipients at our institution.

## Materials and methods

The Mayo Clinic Institutional Review Board approved the study and waived informed consent due to minimal risk. We queried our prospectively maintained institutional transplantation registry to identify adult (≥18 years of age) lung transplant recipients from 2001 to 2015 who had a documented clinical or laboratory diagnosis of postoperative chylothorax, defined as occurring within two months after transplantation. Heart-lung recipients were excluded from the study. A retrospective review of the electronic health records of these patients was performed. Data variables collected included demographics, indication for transplantation, surgical approach and type of transplant (unilateral or bilateral), management and complications of chylothorax. Time to resolution of chylothorax was defined as the time (in days) from the first diagnosis of chylothorax to the removal of the last chest drain without the need for additional drainage. JMP® Pro 10.0.0 statistical software was used to estimate survival curves of the cohort with chylothorax and the rest of the lung transplant population at our institution using the Kaplan-Meier method; the survival curves of the two groups were then compared using the log-rank test method. Survival times are reported as mean (standard error) and median values. Statistical significance was accepted as *P *< 0.05.

## Results

During the study period, postoperative chylothorax was documented in seven (1.38%) out of 504 lung transplantations that were performed from 2001 to 2015. These included four (57%) men and three (53%) women with a median age of 58 years (range: 37-67 years). The most common indication for transplantation was pulmonary fibrosis (five patients; 71.4%). All patients excepting one received bilateral lung transplantation. One patient underwent bilateral re-transplantation for graft failure one year after previous bilateral lung transplantation. The average recipient body mass index (BMI) was in the overweight range at 27.2 ± 4. Patient demographics and chylothorax characteristics are shown in Table 1. 

**Table 1:** Patient demographics and characteristics of chylothorax CPB, cardiopulmonary bypass; PLT, posterolateral thoracotomy; LAM, lymphangioleiomyomatosis

Pt No.	Age	Sex	Diagnosis	Lung transplant type	Surgical approach	CPB	Side of chylothorax	Time from transplant to presentation (days)	Resolution time (days)	Survivalin months	Current survival status
1	48	M	Silicosis	Bilateral	Clamshell	No	Bilateral	7	22	189	Alive
2	66	F	Pulmonary Fibrosis	Unilateral [R]	PLT	Yes	Unilateral [R]	35	14	31	Deceased
3	67	M	Pulmonary Fibrosis	Bilateral (retransplant)	PLT	No	Bilateral	30	7	17	Deceased
4	63	M	Bronchiectasis	Bilateral	PLT	No	Unilateral [R]	4	8	91	Alive
5	58	M	Pulmonary Fibrosis	Bilateral	PLT	No	Unilateral [R]	3	7	61	Alive
6	37	F	LAM	Bilateral	Clamshell	Yes	Unilateral [R]	6	Unresolved	27	Alive
7	44	F	Pulmonary Fibrosis	Bilateral	PLT	Yes	Unilateral [R]	35	60	18	Alive

Presentation, management, and outcomes of chylothorax are summarized in Table 2.

**Table 2:** Presentation, management, and outcomes of chylothorax TPN, total parenteral nutrition; NPO, nil per os; LTD, ligation of thoracic duct

Pt. No	Presentation	Diet	TPN?	Total TPN time	Octreotide?	Type(s) of intervention	Final status
1	Chylous chest tube drainage, acute clamshell thoracotomy wound dehiscence	Chylothorax diet before surgery; NPO followed by low fat diet after surgery	Yes	14	No	Reoperation with washout and repair of sternal dehiscence	Resolved
2	General weakness, malnutrition, pleural effusion	Non fat diet	No	N/A	Yes	Thoracentesis, Pigtail thoracic drain	Resolved
3	Extreme fatigue, anorexia, failure to thrive, pleural effusion	NPO then low fat diet	Yes	15	No	Bilateral pigtail thoracic drain	Resolved
4	Chylous chest tube drainage	NPO	Yes	5	Yes	LTD and oversewing of leak	Resolved
5	Chylous chest tube drainage	Chylothorax diet	No	NA	Yes	None	Resolved
6	Chylous chest tube drainage, Pericardial effusion, large volume pleural effusions, Denver shunt thrombus, empyema, chylous ascites	NPO then chylothorax diet	Yes	23	Yes	Attempted TD embolization; Surgery - LTD, pleurodesis, Denver shunt; paracentesis, multiple thoracentesis and drains	Persistent
7	Malnutrition, pleural effusion	Chylothorax diet then NPO; resumed chylothorax after surgery	Yes	31	Yes	Pigtail thoracic drain; attempted TD embolization; Surgery – LTD	Resolved

The majority (five patients; 71.4%) developed only a unilateral right-sided chylous effusion. Chylothorax was diagnosed in the first week after transplantation in four patients who presented with chylous chest tube drainage, while in the remaining three, the presentation and diagnosis were delayed until approximately one month after surgery. One patient developed a milky chest tube output seven days after transplantation and was managed with a chylothorax diet but had acute wound dehiscence of his clamshell thoracotomy incision one week later with chyle draining through the wound. All three patients with delayed presentations were readmitted with failure to thrive and poor appetite a month after transplantation and on workup were found to have large pleural effusions. The chylothorax diagnosis was confirmed by pleural fluid analysis for triglycerides and chylomicrons in two patients, while in the other five, the diagnosis was clinically based on the milky appearance of chest tube effluent.

Management

Non-operative management was initially attempted in all seven patients and resulted in complete resolution of chylothorax in three (42.9%). Successful nonsurgical management included a combination of pigtail catheter drainage, nothing by mouth (nil per os, NPO), total parenteral nutrition (TPN), and subcutaneous octreotide in two patients, while only octreotide and a chylothorax diet (low-fat, medium-chain triglyceride diet) were required in the third patient who already had chest tubes.

The remaining four patients underwent surgery after failure of conservative management (Table 2) and included three elective TD ligations and one emergent operation. The chylothorax successfully resolved after duct ligation in two of the three patients (66.7%) even though only one had an intraoperatively visible site of chyle leak on the TD. The fourth patient underwent emergent surgical exploration for clamshell thoracotomy wound dehiscence, with washout of the pleural spaces and rewiring of the sternum. No TD ligation was performed, and postoperatively, he was managed with two weeks of NPO and TPN before his chylothorax completely resolved. 

TD lymphangiography and embolization were unsuccessfully attempted in two patients, one of whom (patient seven ) had earlier failed both conservative management and surgical ligation of the TD. The second patient subsequently underwent surgical TD ligation with successful resolution of chylothorax. Subcutaneous octreotide was given to five out of seven patients but non-uniform dosing and duration of the drug limit analysis of whether it contributed to the resolution of chylothorax. 

Outcomes

Complete resolution of chylothorax was seen in six (85.7%) patients. Patient seven who underwent bilateral transplantation for lymphangioleiomyomatosis (LAM) failed multiple interventions including open TD ligation, followed by talc and mechanical pleurodesis. She developed a chylous pericardial effusion that required the creation of a surgical pericardial window to the peritoneal cavity. Prolonged TPN, NPO, octreotide therapy, and attempted embolization of the TD were also unsuccessful. Subsequently, she developed chylous ascites and underwent intra-abdominal lymphatics embolization with resolution for one year, which reoccurred, and a peritoneo-jugulovenous (Denver) shunt was placed, which helped control the effusion and ascites and the shunt was removed later. The patient eventually developed sepsis secondary to Staphylococcal empyema. The initial Denver shunt became non-functional due to thrombus and was removed. Her chylothorax recurred and was managed with multiple thoracentesis and large volume paracentesis procedures until the Denver shunt was replaced. She has had multiple admissions since then for various transplant- and chylothorax-related complications. She underwent intra-abdominal lymphatics embolization. In addition, she failed therapy with Sirolimus and Everolimus before she underwent ligation. 

The median time for the resolution of chylothorax was 11 days (range: 7-60 days) and the median length of stay was 55 days (range 9-104 days). The mean survival for patients with chylothorax was 29.2 (3.1) months, compared to 78.2 (2.9) months for the rest of the transplant population (*p* = 0.37). The median survival was not reached for the chylothorax group and was 71.8 months (95% CI: 58.0-83.9) for the remaining patients.

## Discussion

As observed in our study, chylothorax is an uncommon complication after lung transplantation but when present can lead to significant morbidity and prolonged hospital stay. Furthermore, patients with post-operative chylothorax often require additional major interventions including re-operations which add to healthcare costs and increase the risks of further complications. Pre-operatively identifying recipients at higher risk for chylothorax may be helpful in discussing the implications and management of this major complication with the patient and their caregivers. The current scarcity of published data about chylothorax after lung transplantation makes it difficult to understand the true course and impact of this complication in this particular group of patients. The results of our study indicate that long-term survival is not adversely affected in patients with post-operative chylothorax.

The mechanism of chylothorax after lung transplantation is probably multifactorial. Uncomplicated lung transplant operations do not usually involve dissection along the normal course of the TD, and hence direct intraoperative injury to the TD is uncommon but may occur in cases where prior infection or pleurodesis obliterates anatomical planes. All the patients in our study were reported to have severe pleural adhesions in their operative notes, placing them at a higher risk of chylothorax. Other plausible reasons for post-transplant chylothorax may include injury to an aberrant TD or lymph leak from disruption of dilated perihilar or mediastinal lymphatics and lymph nodes [9]. Partial or complete obstruction of superior vena cava or subclavian veins due to long-standing indwelling central venous catheters raises the pressure in the lymphatics, thereby making them fragile and increases the risk for chyle leak. Lymphangioleiomyomatosis (LAM) is another condition with a high risk for chylothorax because of the extensive network of lymphatics that are present within the chest in these patients; the reported incidence of post-transplant chylothorax is around 7% to 8% in LAM patients [10-12]. In a previously published study of 12 patients with LAM who underwent lung transplantation at our institution, one patient (8.3%) developed post-operative chylothorax [13]. As described above, chylothorax in our patient with LAM has been the most challenging to manage, having failed multiple interventions and currently controlled with Denver shunt. 

**Figure 1:**
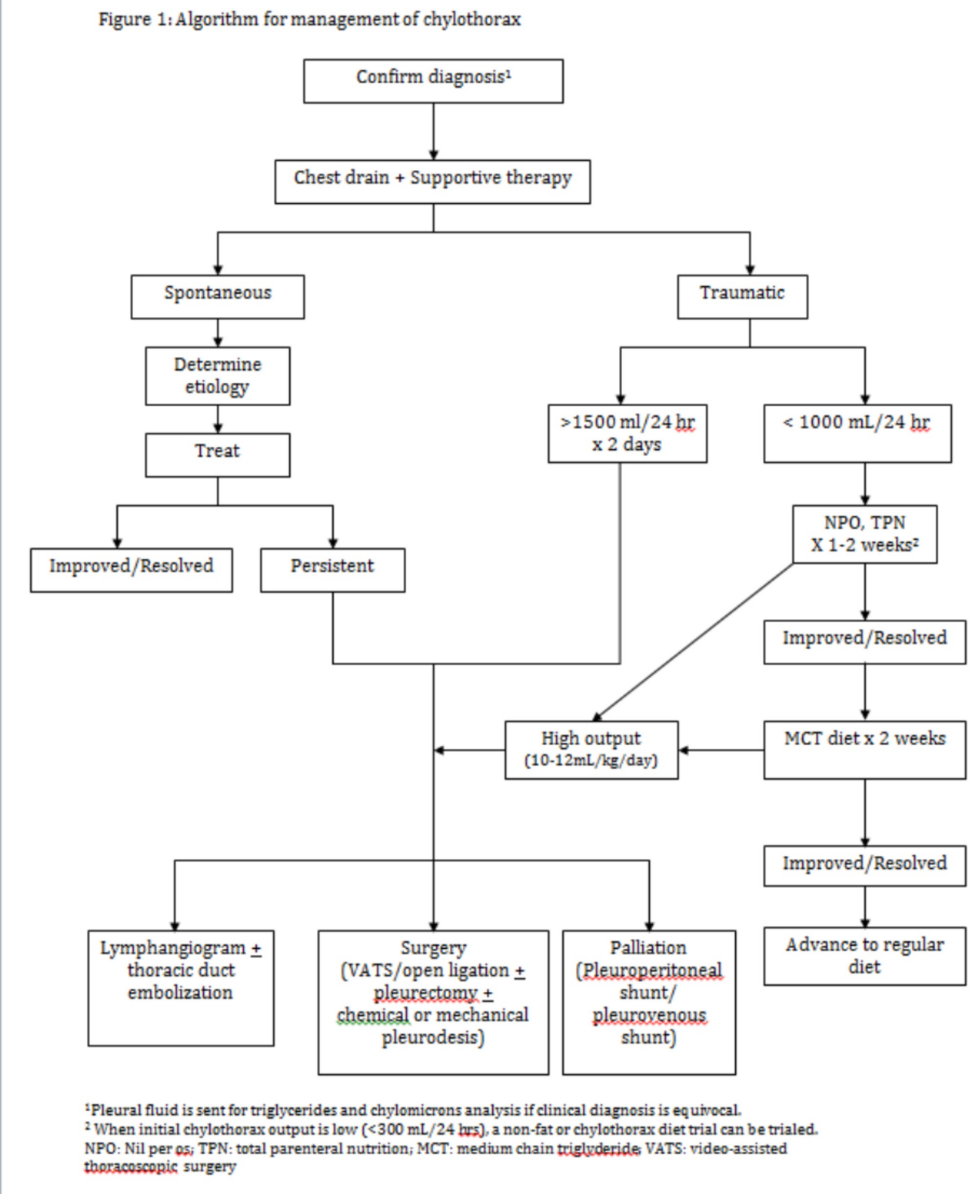
Algorithm for management of chylothorax ^1^Pleural fluid is sent for triglycerides and chylomicrons analysis if the clinical diagnosis is equivocal. ^2^When initial chylothorax output is low (<300 mL/24 hours), a non-fat or chylothorax diet trial can be trialed. NPO, nil per os; TPN, total parenteral nutrition; MCT, medium-chain triglyceride; VATS, video-assisted thoracoscopic surgery

Post-operative chylothorax can be extremely challenging to manage but resolved in about 40% of patients in our study who were managed initially with non-operative methods. Adequate chest drainage and prevention of dehydration, electrolyte imbalance, malnutrition, and infections are the goals of non-surgical management [14]. Both supportive and directed therapies are essential in ensuring good outcomes in these patients who are often debilitated and immunocompromised. Our current algorithm for management of post-operative chylothorax is shown in Figure 1.

Chylothorax is often diagnosed based on high volume chest tube output with a milky appearance of pleural fluid. Chemical analysis of the pleural fluid for triglycerides and chylomicrons is performed only if there is any doubt about the diagnosis. We considered persistent high output (average daily volume: >1500 mL/day for two days or >1000 mL/day for two days) in the early post-operative period or after a longer period of non-surgical therapy (average daily volume: 10-12 mL/kg/day for two weeks) as an indication for active intervention [3,15]. A prior study of post-operative chylothorax in non-transplant patients showed 49% success with non-surgical management [1]. Surgical ligation of the thoracic duct has been historically considered the definitive treatment for post-operative chylothorax that fails conservative management and has been reported to be exceptionally successful [15-16]. TD embolization is a less invasive alternative to surgical ligation but could not be successfully performed in the two patients in our patient cohort due to difficulties in canalizing the duct. Other authors have reported successful clinical resolution of chylothorax in 72% to 90% of patients with embolization when the TD can be canalized [17-18]. Other surgical options include mechanical and chemical pleurodesis or even a parietal pleurectomy. Pleuroperitoneal and peritoneovenous shunts are palliative therapies that provide a pathway for recycling the chylothorax into the peritoneal cavity or venous system. In patients with chylous ascites, the pleuroperitoneal shunt is contraindicated. Pleurovenous shunts have been reported to be successful in a few cases of refractory chylothorax by other authors [12,19-20]. As noted in the LAM patient in our study, peritoneovenous or pleurovenous shunts may occasionally fail requiring replacement.

Our study has certain limitations, which include its retrospective nature even though the data were collected prospectively. It is possible that minor chyle leaks may have occurred in other lung recipients and may have spontaneously stopped prior to being clinically detected. As a heterogenous group, lung transplant recipients have multiple comorbidities that may confound the true course of post-operative chylothorax. Treatment and timing of interventions were not uniform in all patients and this makes it challenging to make strong recommendations regarding the management of chylothorax except to suggest that conservative management still plays a significant role in initial management. Although the number of patients with chylothorax in our study is small, it provides an important perspective into an uncommon but potentially devastating complication that to the best of our knowledge has not been analyzed before in a large, single institutional group of lung transplant recipients. 

## Conclusions

Chylothorax is a rare complication after adult lung transplantation that can lead to major comorbidities and prolonged hospital stay. When managed appropriately, it has no significant effect on long-term survival. Nonsurgical management should be attempted initially and may be successful in up to 40% of patients. Surgical ligation of the thoracic duct should be considered in patients who have failed conservative management. 
